# Differentiating Oneiric Stupor in Agrypnia Excitata From Dreaming Disorders

**DOI:** 10.3389/fneur.2020.565694

**Published:** 2020-11-12

**Authors:** Luca Baldelli, Federica Provini

**Affiliations:** ^1^Department of Biomedical and Neuromotor Sciences, University of Bologna, Bologna, Italy; ^2^Istituto di Ricovero e Cura a Carattere Scientifico Istituto delle Scienze Neurologiche di Bologna, Bologna, Italy

**Keywords:** oneiric stupor, agrypnia excitata, dreams, sleep, differential diagnosis, RBD, nightmares, hallucinations

## Abstract

Oneiric Stupor (OS) in Agrypnia Excitata represents a peculiar condition characterized by the recurrence of stereotyped gestures such as mimicking daily-life activities associated with the reporting of a dream mentation consisting in a single oneiric scene. It arises in the context of a completely disorganized sleep structure lacking any physiological cyclic organization, thus, going beyond the concept of abnormal dream. However, a proper differential diagnosis of OS, in the complex world of the “disorders of dreaming” can become quite challenging. The aim of this review is to provide useful clinical and videopolygraphic data on OS to differentiate it from other dreaming disorders. Each entity will be clinically evaluated among the areas of dream mentation and abnormal sleep behaviors and its polygraphic features will be analyzed and distinguished from OS.

## Introduction

As the search for an understanding of dreaming is more than thousands of years old, scientific evidence developed over the last century (1, 2). Several biological and psychological theories about the purpose of dreaming have been put forward (3) as recent theories suggest that dreams fulfill an adaptive function related to emotion-regulation, learning, and memory consolidation (4, 5), but also constitute a biological defense mechanism, evolved as a capacity to repeatedly simulate threatening situations (6). On the other hand, while sleep medicine has greatly developed in the last decades, the phenomenology and physiology of dreaming have been overlooked, despite their relevance, by sleep specialists. In fact, objective analysis of dreams is very difficult (7), requires deep neuroscientific knowledge (8), and most of the information comes from the patients' reports, while, thanks to polygraphic recordings and imaging, dream correlates have been studied deeply (9). The identification and videopolysomnographic (vPSG) studies of several neurological and even severe disorders associated with abnormal dreaming or dreaming-like events have provided valuable knowledge on conditions such as Rapid Eye Movement (REM) Sleep Behavior Disorder (RBD), Nightmare Disorder, Disorders of Arousal (DoA) and sleep-related hallucinations. Among neurological disorders, Oneiric Stupor (OS) in Agrypnia Excitata (AE) represents a peculiar condition, arising in the context of a deranged sleep structure lacking of any physiological cyclic organization, thus, going beyond the concept of abnormal dream (10).

However, a proper differential diagnosis of OS, among the complex world of the “disorders of dreaming” can become quite challenging, especially when patients report the so called “bad dreams” and “agitations during night.”

The aim of this review is to deliver useful data on OS in a clinical and videopoligraphic approach, to differentiate it from abnormal dreaming. Each entity will be clinically evaluated among the areas of dream mentation and abnormal sleep behaviors (e.g., dream enactment) and its polygraphic features will be analyzed and distinguished from OS (see Table 1 summarizing the different features). Finally, brief remarks on physiopathology and treatment will be given when appropriate.

**Table 1:** Differences between oneiric stupor and the other described conditions.

**Feature**	**Oneiric stupor**	**RBD**	**Nightmare**	**Sleep-related hallucinations**	**Lucid dreaming**	**Disorders of arousal**
Timing	Throughout the 24 h.	At least 60–90′ after sleep onset. Usually in the latter part of the night.	Usually in the latter part of the night.	Hypnagogic: at sleep onset. Hypnopompic and complex: awakening at late night or in the morning.	Usually in the latter part of the night.	First third of the night.
Stage	Wake, subwakefulness (mixed EEG state with features of both N1 and REM sleep), REM.	REM	REM. NREM also in post-traumatic nightmares.	Hypnagogic: sleep-wake transition. Hypnagogic in sleep deprivation and narcolepsy: SOREMPs. Hypnopompic: REM intruding into wakefulness. Complex: N2-N3.	REM	N3
Sleep structure	Completely disorganized	Normal. REM sleep without atonia.	Normal. Fragmented by awakenings.	Normal	Normal	Normal
Duration	Minutes	Tens of seconds	Up to many minutes	Seconds-Minutes	Tens of minutes, up to an hour	Minutes
Frequency (per night)	Continuous or sub-continuous state	Usually 1–4 per night	One to many per night	Usually one per night	Usually one per night	Usually one per night
Motor features	Quiet, stereotyped, and repetitive gestures usually mimicking daily-life activities. Influenced by patient routine or hobbies.	Violent behaviors and vocalizations mimicking the content of the dream, including punching, or kicking. Non-violent elaborate behaviors may also occur.	None	None. Muscle paralysis can be associated.	None	Variable: exploring environment, manipulating objects usually with eyes opening, fixing covers, screaming, speaking, sleepwalking.
Mentation content	Single “oneiric scene.”	Complex “dream tale.” Dream content usually involve (active) defense against aggression.	Themes related to fear and involving a direct threat to mental/physical integrity.	Single or multisensory. Visual: kaleidoscopically phenomena, light flashes, lifelike images. Auditory: voices, steps. Somatic: body distortions, entities climbing over the body.	Highly variable. Awareness of the dreaming state associated with intentional performing of waking life actions.	Full dream recalls usually absent, with variable content.
Emotional load	No emotional involvement.	High. Especially fear and anger.	Very high. Fear, anger, sadness, helplessness, anxiety and frustration.	Usually very high. Unpleasant and frightening.	Low	Variable. From frightening to emotionally neutral.
Bizarreness	Not bizarre	Usually not bizarre. Sometimes unusual situations are possible (e.g., attacked by exotic animals).	Very bizarre	Very bizarre	Bizarre	Bizarre
Spatial reference	Self-centered. The patient is usually the only protagonist of the oneiric scene.	Self-centered. The actions performed are usually hetero directed.	Self-centered.	Outside the subject. Images, sounds, silhouettes have no or little interaction with the subject.	Self-centered.	Self-centered.
Focalization^*^	(11)	External	Internal	Internal	Internal	Zero	Internal
Autonomic activation	Marked autonomic hyperactivity.	Blunted, tachycardia may not accompany the impressive movements.	Accelerated heart and respiratory rates usually precede the awakenings.	Accelerated heart and respiratory rates when frightening.	Higher autonomic activation typical of active REM.	Marked autonomic activation.

* *The term focalization, used in modern narratology, describes the kind of perspective from which the events of a story are witnessed. The term “zero focalization” corresponds to an omniscient narrator, where the narrator knows more than the character, or more exactly, says more than any of the characters knows: Narrator (the dreamer) > Character (the subject of the dream). In “internal focalization,” the narrator knows/says only what a given character knows (Narrator = Character). In the third case “external focalization,” the narrator knows/says < the character knows (Narrator < Character)*.

## Oneiric Stupor in Agrypnia Excitata

### Definition

The term agrypnia excitata (*agrypnia* meaning “chasing sleep away” referring to sleep loss of organic origin, and *excitata* referring to the motor and autonomic activation) defines a generalized overactivation syndrome characterized by severe and persistent insomnia and marked motor and autonomic sympathetic activation (12, 13). Peculiar episodes of Oneiric Stupor characterize AE with the recurrence of stereotyped gestures such as mimicking daily-life activities associated with the reporting of a dream mentation consisting in a single oneiric scene (13).

### Associated Clinical Conditions

AE has been described in separate rare clinical conditions implying a thalamo-limbic system dysfunction. Fatal Familial Insomnia (FFI), a human prion disease characterized by severe but selective atrophy of thalamic mediodorsal and anteroventral nuclei with disconnection of the limbic cortical areas and subcortical regions, represents the first and most known condition (14, 15). Morvan Syndrome (MS), an autoimmune limbic encephalopathy, and Delirium Tremens (DT), the well-known alcohol withdrawal syndrome (16, 17), determine, instead, a functional interruption of the thalamo-limbic circuits which regulate the sleep–wake cycle and the control of the autonomic system (12, 18). AE can also arise from other conditions such as Whipple's Disease (19) and is anecdotally described in Creutzfeldt-Jakob Disease and Mulvihill-Smith (20, 21).

### Clinico-Polygraphic Features for Diagnosis

**Figure 1:**
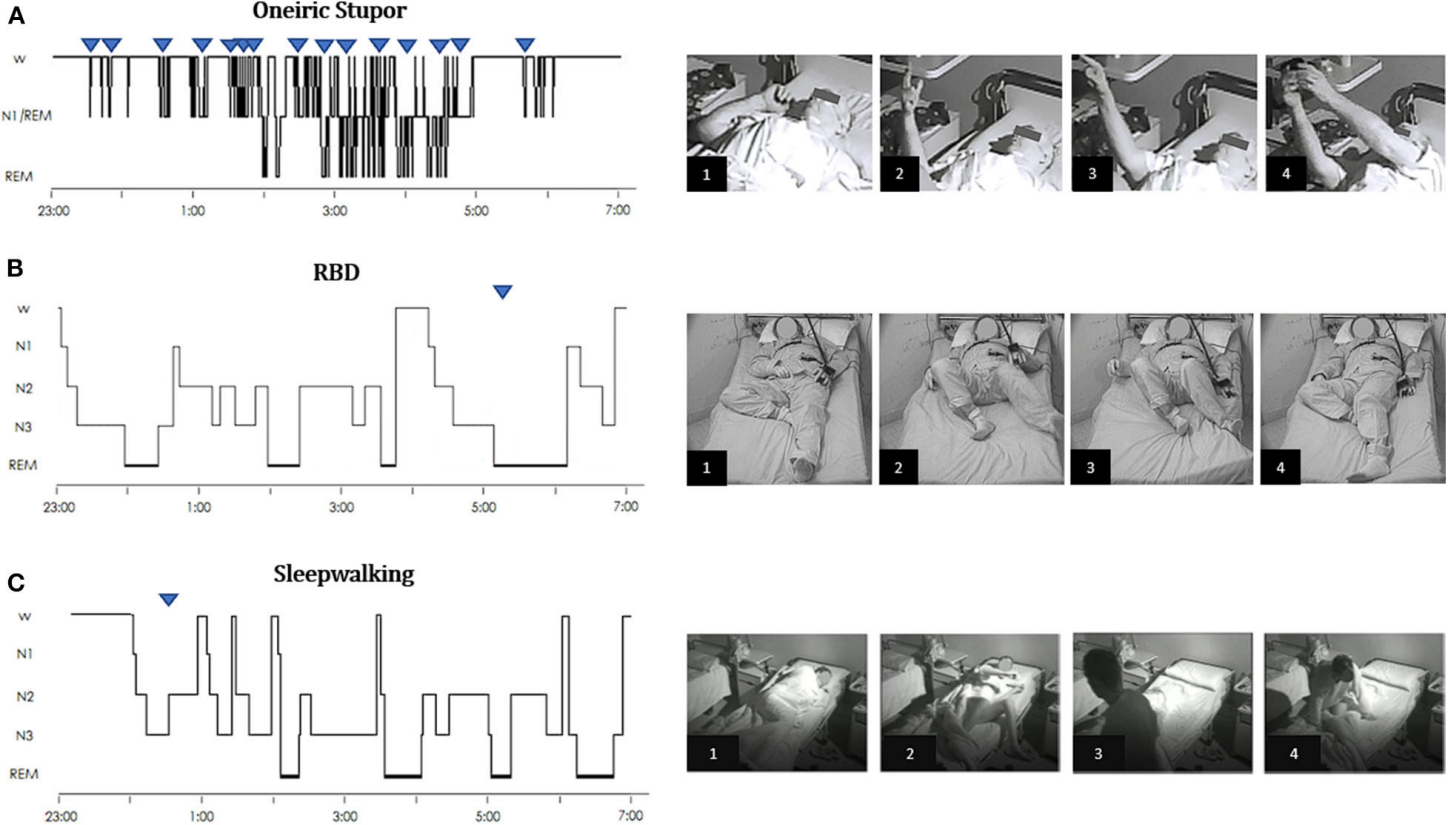
Hypnograms and frame sequences of Oneiric Stupor (OS) vs. parasomnias. **(A)** Oneiric Stupor. The hypnogram continuously fluctuates between wake and subwakefulness (N1/REM) with short intrusions of REM sleep; episodes of OS arise subcontinuously during wakefulness when the patient is left alone, subwakefulness or REM sleep. During each episode, the patient performs gestures such as pointing at something and manipulating an inexistent object, quietly mimicking usual daily life activities. **(B)** RBD. The hypnogram shows a physiological sleep structure. In the recorded episode, arising from REM sleep without atonia, the patient starts to move the legs and suddenly kicks out of the bed with his left leg as if he was targeting a specific object. When questioned about the dream content the patient reported that he was cycling and one person would chase him by bicycle, so that when the pursuer reached him, the patient tried to knock the pursuer off his bike by kicking the spokes of the wheel. **(C)** Sleepwalking. The hypnogram shows a physiological sleep structure, with some infrasleep awakenings. Arising from NREM sleep, during the episode the patient gets up, starts walking in the room, and finally turns in bed. Upside-down triangles represent recorded episode(s) during nocturnal videopolysomnography. [Modified with permissions from (23) and (24) under Creative Commons Attribution 4.0 International License—http://creativecommons.org/licenses/by/4.0/].

In OS, patients, when left to themselves, may lapse into typical episodes of dream enactment, whereby they perform movements mimicking the contents of their dreams (often daily life activities). The same gestures recur in different patients, such as combing the hair, dressing, washing, eating, and drinking. Patients are able to recall upon “awakening,” if asked, as a single oneiric scene [(22); Figure 1A]. With the progression of the disease, (especially in patients with FFI), recall of the mental content becomes hard or impossible, and patients become progressively more confused, alternating between wakefulness and oneiric confusional states (22). However, for a proper diagnosis of OS in the context of Agrypnia Excitata, other two clinico-neurophysiological conditions must be demonstrated: (1) slow wave sleep loss with disruption of the physiological sleep-wake cycle, (2) day and night, motor, sympathetic and aminergic overactivity (12, 18, 25–27).

**Figure 2:**
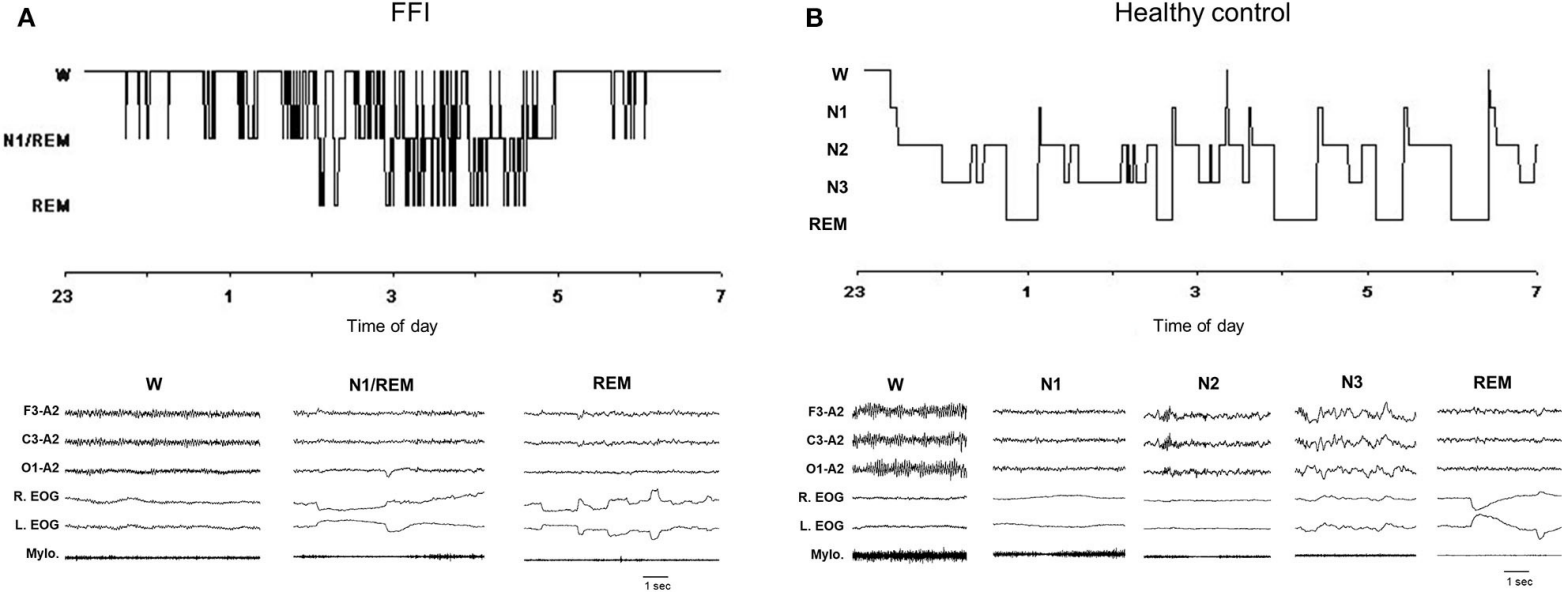
Hypnogram (upper graph) and related excerpts of a polygraphic tracing (lower graph) in a patient with Fatal Familial Insomnia (FFI) **(A)** and in an age-matched healthy control individual **(B)**. In the FFI patient hypnogram continuously fluctuates between wake and subwakefulness (N1/REM) with short intrusions of REM sleep; the polygraphic excerpts show abolishment of spindle and delta sleep. EEG (F3-A2; C3-A2; O1-A2); R, right; L, left; EOG, electrooculogram; Mylo, mylohyoideus muscle [From (23), with permissions].

Disruption of the sleep-wake cycle consists in: (a) the disappearance of spindle-delta activities (Slow Wave Sleep—SWS), (b) failure in REM sleep stabilization, with REM appearing only in short recurrent episodes or mixed with stage 1 Non-REM (NREM) sleep. Longitudinal 24-h polysomnographic monitoring documents mainly two alternating states (28, 29). The cyclic structure of sleep is lost, and short periods of REM sleep can also arise from a state of wakefulness, associated with absent or slightly reduced muscle atonia (28, 29). A state of “subwakefulness” is therefore the predominant nocturnal and diurnal EEG and behavioral pattern consisting of stage 1 NREM sleep interrupted by sudden-onset episodes of rapid-eye-movement sleep with or without atonia lasting a few seconds or minutes [(26); Figure 2]. This condition represents the neurophysiological substrate of dream enacting behaviors which characterize oneiric stupor, emerging from any of these stages.

Finally, 24-h diurnal and nocturnal (circadian) sympathetic and aminergic overactivity are fundamental signs of AE. Of significance is the steady higher secretion of noradrenalin (NA) during both day and night in opposition to reduced melatonin (MLT) levels and the absence of its nocturnal peak, the latter could constitute a biological marker of AE (26).

### Physiopathology

Spindles and slow wave sleep invariably disappear in AE, independent of its primary cause as the thalamus is the structure most severely impaired or damaged and thalamo-cortical and cortico-thalamic circuits are deeply involved by the pathological processes (26). The thalamus has a fundamental role of connection for the structures governing slow-wave sleep onset and continuity by means of its mediodorsal nucleus allowing extra thalamic connections for the reticular nucleus (30, 31), original generator of sleep spindles (32). Nevertheless, REM sleep continues to be present or even becomes overrepresented (as in DT) because its pontine generator is undamaged and transmission of the signal originating from the REM-on system to the forebrain follows extrathalamic pathways, as originally proposed by Jouvet (33) and confirmed since (34, 35).

### Prognosis and Treatment

Prognosis and treatment deeply vary on the original disorder causing AE and OS episodes. Regarding FFI, as with all human prion diseases, there is no currently available treatment or cure (36); in FFI patients AE does not respond to the typical symptomatic treatment of insomnia such as sedatives and benzodiazepines (37, 38). On the contrary clonazepam (up to 4 mg per day) seems to be effective in reported cases of AE in DT (17). Finally, the removal of MS causative VCKC antibodies by means of plasma exchange also improves AE and episodes of OS, although maintaining a poor prognosis (16, 39). While MS patients can recover up to a full resolution (39–41) and DT is usually a transient condition, the presence of a fully developed AE usually implies worse prognosis (12).

## REM Sleep Behavior Disorder

### Definition and Diagnostic Criteria

RBD is a REM sleep parasomnia characterized by abnormal behaviors emerging during REM sleep. These RBD behaviors manifest as an enactment of frequently unpleasant, violent and full of action dreams, causing sleep disruption and even injuries to the patient or to their bed partner (42). RBD is also associated with electromyographic abnormalities during REM sleep, the electromyography (EMG) demonstrating an excess of muscle tone, and/or an excess of phasic EMG twitch activity during this phase (42). According to the International Classification of Sleep Disorders (ICSD-3) the diagnosis of RBD consists of all the following criteria: (a) repeated episodes of sleep related vocalization and/or complex motor behaviors; (b) these behaviors are documented by polysomnography to occur during REM sleep or, based on clinical history of dream enactment, are presumed to occur during REM sleep; (c) polysomnographic recording demonstrates REM sleep without atonia (RSWA); (d) the disturbance is not better explained by another sleep disorder, mental disorder, medication, or substance use (42).

### Associated Clinical Conditions

RBD can be idiopathic or associated with neurologic disorders, mostly neurodegenerative diseases such as α-synucleinopathies with different prevalence. Parkinson's disease (PD) patients present an RBD prevalence of about 25% (43). Moreover, the sleep disorder is so frequent in Dementia with Lewy Bodies—DLB (up to 76% of patients) to have become a core feature for its diagnosis (44, 45); RBD also affects almost the totality of Multiple System Atrophy patients (46). RBD can also be associated with neurological diseases with autoimmune etiology such as narcolepsy or Anti-IgLON5 disease (47), with specific brain lesions (48) or be consequent to drug/substance intake or discontinuation (49).

The actual definition of idiopathic RBD (iRBD) has been recently reconceived after increasing evidence showed that the vast majority of patients with iRBD develop a neurodegenerative disease over time, of the class of alpha-synucleinopathies (50). The risk of conversion is over 80% in longitudinal studies with a follow-up of over 10 years, with a yearly conversion rate of 6.3% (51–53). Moreover, a recent study showed that even in iRBD patients who did not convert after a follow-up of 10 years or more, biomarkers of a-synuclein–related neurodegeneration are present (54). Considering these data, iRBD is nowadays conceived an early stage alpha-synucleinopathy. Consequently, the term “isolated” RBD has been proposed as more appropriate, reflecting the spectrum of progression of the disorder as a continuum (55).

### Epidemiology

The most precise estimates of iRBD prevalence come from community- based vPSG studies. One study demonstrated a prevalence of 1.15% for in South Korea (56) and a second study (HypnoLaus) reported a similar prevalence of 1.06% for RBD by means of dynamic at home vPSG (57). Interestingly, no clear difference of prevalence was shown between men and women, suggesting that the strong male pre-dominance, usually observed in sleep clinics, probably comes from a selection bias related to RBD in males being more aggressive and violent and therefore more clinically reported than RBD in females (57).

### Clinical Features for Diagnosis

Abnormal dream enactment behavior more frequently consists in motor episodes and nocturnal vocalizations. Motor episodes present with a wide spectrum of intensity and complexity, varying from mere muscular twitching to complex and elaborate movements [(58), Figure 1B]. Single or repeated limb or truncal twitches constitute the most frequently observed motor manifestations and could be indistinguishable from startled reactions (59). However, the enactment of a dream with aggressive content is most typically present, such as punches, kicks, and blows are reported in more than 80% of patients resuming in some cases in actual episodes of violent behavior (unwillingly) directed to the bedpartner (58). These episodes usually present with a sudden start, last few seconds or minutes and, in the majority of cases, are spatially limited within the bed, even if leaping and falling from bed is also reported (60). It is exactly from these behaviors that personal lesions (and to the bedpartner) can happen, such as bruises, but also wounds, joint dislocations, fractures, and even subdural hematomas (50, 61). Non-violent but still complex behaviors are also possible, such as eating, playing sports, or even dancing in bed, etc. (59). Most behaviors are learned behaviors in accordance with the cultural and social context of the patient (62). The most frequent vocalizations manifest as screaming, yelling, and groaning but are also organized in intelligible words and even phrases as in actual arguing. Less frequently, equally complex, but non-aggressive vocalizations are produced, such as crying, laughing, whistling, singing, giving a speech, etc. (59). Some authors emphasized that it is possible to underline a motor signature of RBD independent of the context generating the disorder (isolated vs. secondary). In contrast with wakefulness, movements during RBD are self-centered and rarely involve the environment (if so, the use of the environment is inappropriate): the patient interacts with the dream's scenario and not with the real one (63).

Altered dream mentation usually manifests in violent or stressful situations such as fighting, arguing, and being chased by an imaginary aggressor, usually unfamiliar people (e.g., strangers with a blurred face) or frightening animals. Patients are always involved in the dream, fighting back vigorously defending themselves or protecting their loved ones from a physical attack. Dreams often contain settings and activities related to the patients' past (58). Patients usually have no history of aggressive or violent behavior during the daytime, but are by contrast more frequently calm and placid (50). In parallel with behaviors, non-violent dream content can also occur. RBD associated dreams are described as vivid, intense, and different from the ones reported before the disorder started (62). Dream recall, especially if collected on the next morning, is frequently precise and well-detailed, even if non-recallers can be present also among RBD patients (64).

Clinically, evoking a clear history of dream enactment behavior can be difficult, as up to 50% of patients might not be aware of the behaviors and/or remember the associated dream content precluding the reporting of a positive history (58). A semistructured interview evaluating both abnormal dream mentation and behaviors/vocalization, especially with the help of the bedpartner, is often necessary for a clinical diagnosis of RBD (60). The concordance between the dream actions (reported upon awakening from the behaviors) and the behaviors observed by the bed sharer or by clinicians on the video-monitoring during proven REM sleep in the sleep lab is called isomorphism (62).

### Polygraphic Features for Diagnosis

The definitive diagnosis of RBD requires a vPSG assessment documenting the presence of EMG REM sleep without atonia as defined by the ICSD-3 and the American Academy of Sleep Medicine (AASM) scoring manual (42, 65). Features essential for the RBD diagnosis include an excess of muscle tone and/or an excess of phasic EMG twitch activity during REM sleep (42). RSWA has a high night-to-night stability (66); accordingly, a single night of vPSG is usually sufficient for the diagnosis of RBD, provided that sufficient REM sleep is present during the recording (usually more than 10% of the total sleep time) (67).

One of the difficulties in polysomnographic (PSG) recordings of patients with RBD is the diagnosis in absence of any recorded RBD episode, based only on RSWA and suggestive history. A global consensus is not present on this topic, but various groups proposed different minimal RSWA values to be present, comprising mentalis tonic EMG activity and/or limbs phasic EMG activities, ranging from 15 to 35% of the total (68). In contrast to the excessive motor activities observed during REM sleep in patients with RBD, all other features of REM sleep, are usually preserved (69). However, periodic leg movements in sleep are described in up to three quarters of patients with RBD (70).

### Physiopathology

At the neuroanatomic level, sublaterodorsal tegmental nucleus (neurons specifically activated during REM sleep send descending efferents to glycine/GABA neurons within the ventral medulla inhibiting spinal motoneurons thus achieving muscular REM atonia. The activation of specific areas on the limbic cortex, retrosplenial cortex in particular, is responsible for the production of oneiric scenarios and for the activation of the motor cortex, responsible for the consequent activation of spinal motoneurons, which are, however, inhibited by GABA- and glycinergic projections (71). In RBD patients, the neurodegeneration of SLD and VM releases the inhibition of spinal motoneurons, impeding the generation of muscular atonia (50). These are the mechanisms underlying RSWA, which is a necessary but not sufficient condition for RBD to develop (62). The disinhibition of mesencephalic motor pattern generators and consequently their phasic activation during REM sleep, leads to the generation of the typical motor behaviors enacting oneiric content (72). The concomitant dysfunction of the limbic system (involved in emotional processing and intensely activated during REM phase) and of the amygdala, in particular, is considered the reason of altered dream mentation intruding into abnormal dream enactment (71). However, the theory of RBD consisting in “acting out dreams” has been challenged by the hypothesis that dream mentation could be actually built up on the abnormal “dreaming out acts” (73). As a matter of fact, Blumberg and collaborators suggested that oneiric contents in RBD could be built up by means of sensory feedbacks from the sensory cortex. Sensory feedbacks normally follow the motor cortex commands during wake, allowing the regulation of the motor program, however, in absence of proper feedback signaling during REM phase, the sensory cortex could integrate these feedbacks into an oneiric scene (74, 75).

### Differential Diagnosis

At first sight, OS bears some resemblance to RBD, regarding dream enactment and REM sleep features of AE. However, the two entities are clearly different. First, RBD arises from a normal sleep–wake cycle as the only alteration is the lack of REM muscular atonia. OS arises within a severely altered sleep structure with a profound loss of slow-wave sleep and a pre-dominance of a mixed state with features of both stage 1 NREM and REM sleep (49). For this reason, OS episodes are not restricted to the latter part of the night like RBD but occur throughout the day and night due to the loss of physiological sleep structure. As a matter of fact, OS tends to present in clusters or sub-continuously if the patient is left alone and not stimulated, whereas RBD usually more frequently occurs once per night. OS lasts longer (up to several minutes) compared to RBD, whose duration does not usually exceed the minute (22). Regarding dream content another difference can be underlined as the content of OS is more likely a simple oneiric scene, usually emotionally neutral with low volition involved, whereas RBD is the enactment of a true REM dream in which emotions and memories are mixed into a fantastic, movie-like plot (76, 77).

### Therapeutic Principles

The primary aim of RBD management is to prevent patient's and bedpartner's injuries due to the abnormal behavior during REM sleep, eventually limiting sleep interruptions or even sleep phobia (55, 78). First, ensuring the safety of the sleep environment should be taken into consideration. Any potentially dangerous item that could be picked up, swung, or thrown should be removed, including routinely used objects like alarm clocks and lamps. Other options to minimize potential injuries could include sleeping bags or bed rails to prevent the patient from falling from the bed or even removing the box spring and bedframe to sleep on the plain mattress (78). Secondly, an adequate pharmacological therapy should be administered, consisting in clonazepam and/or melatonin for most of the patients. Both medications are effective and relatively well-tolerated and retain strong efficacy over time (79).

## Sleep Related Hallucinations

### Definition

Sleep related hallucinations are hallucinatory experiences that occur at sleep onset or on awakening from sleep. They are pre-dominantly visual but may include auditory, tactile, or kinetic phenomena (42). Three categories of sleep related hallucination can be distinguished following ICSD-3 categorization (42, 80). Hallucinations at sleep onset (*hypnagogic hallucinations*), which could be of difficult differentiation from sleep onset dreaming. Hallucinations on waking, especially late at night or in the morning, (*hypnopompic hallucinations*), which may arise from a period of REM sleep with patients being uncertain on their waking or dream-related origin. *Complex nocturnal visual hallucinations* represent a distinct form of sleep related hallucinations (81). They typically occur following a sudden awakening, without recall of a preceding dream. They usually take the form of complex, vivid, relatively immobile images of people or animals, sometimes distorted in shape or size. These hallucinations may remain present for many minutes but usually disappear if ambient illumination is increased (82).

### Associated Clinical Conditions

#### Hallucinations as Isolated Phenomena

Isolated fleeting perceptual experiences occurring in the wake-sleep transition (hypnagogic hallucinations) and from sleep to wakefulness (hypnopompic hallucinations) are found in up to 70% of the general population (83). They are involuntary, spontaneous and of varying emotionality (84). Both hypnagogic and hypnopompic hallucinations are more common in children and young adults and slightly more prevalent in women than in men (83). Conversely, complex nocturnal visual hallucinations appear to be much rarer in the isolated form, occurring in the setting of a range of neurologic and visual disorders (see following paragraphs) (81). ICSD-3 criteria include: (a) a complaint of recurrent hallucinations that are experienced just prior to sleep onset or upon awakening during the night or in the morning, (b) their prominent visual nature, and (c) the disturbance is not better explained by another sleep, mental or medical disorder or by medication or substance use (42).

#### Hallucinations Related to (Recurrent) Isolated Sleep Paralysis

Sleep paralysis consists in transient, generalized, inability to move and to speak occurring in the transitional period between wakefulness, and sleep (85). It is characterized by the complete inability to move in a subjectively awake person (86). It affects all somatic voluntary muscles except for diaphragm, extraocular muscles, and the stapedius, similar to the physiologic muscle atonia experienced during REM (87). Sleep paralysis can last up to several minutes (88), disappearing spontaneously or by means of an external stimulation. It is frequently associated with hypnopompic hallucinations (89, 90). The experience of a full-body paralysis despite subjective alertness is overwhelmingly unpleasant and frightening, especially when occurring for the first time.

The diagnostic criteria for Recurrent Isolated Sleep Paralysis, include: (a) recurrent inability to move the trunk and all of the limbs at sleep onset or upon awakening from sleep; (b) each episode lasts seconds to a few minutes; (c) episodes cause clinically significant distress including bedtime anxiety or fear of sleep; and (d) the disturbance is not better explained by another sleep, mental or medical disorder or by medication or substance use (42).

#### Hallucinations in Narcolepsy

Hypnagogic and hypnopompic hallucinations (as well as complex nocturnal hallucinations) and sleep paralysis, can act as elements of the clinical tetrad of narcolepsy (i.e., sleep attacks, cataplexy, hypnagogic hallucinations, and sleep paralysis), where they have been reported in as many as 50% of patients (91). Usually, patients realize immediately afterwards that the experiences are not real, but the hallucinations in narcolepsy can be severe enough to mislead to an incorrect diagnosis of schizophrenia (92). Hallucinations in narcolepsy must be differentiated from dream delusions: a condition where narcoleptic patients act according to previously experienced dreams (93).

#### “Sleep Related” Hallucinations Not Actually Related to Sleep: Dementia With Lewy Bodies and Charles Bonnet Syndrome

In patients with Dementia with Lewy Bodies, but also with longstanding Parkinson's Disease, nocturnal hallucinations are part of a greater clinical picture present also during the day and are not clinically and polygraphically associated with sleep (94). The patient is awake during these episodes but could be poorly responsive and disoriented due to the underlying neurodegenerative condition (95). Indeed, a number of factors may explain the relationship of the hallucinations to night time, such as their typical occurrence at the end of the day where low ambient illumination may play a role and images can vanish upon switching on the light (81). Complex visual hallucinations are the predominant manifestation, although auditory hallucinations and hallucinations of sensed presence can also happen (80). Visual hallucinations often comprise formed complex percepts (e.g., people, faces, animals, and objects), or, less commonly, simple percepts (flashes and dots) (96). Hallucinations in DLB and PD are usually perceived to be real and unpleasant, but not frightening (97). Finally, visual hallucinations phenomenologically similar to hypnagogic hallucinations (simple hallucinations such as shapes, lines, colors, etc…), but also to complex visual hallucinations, can occur in otherwise healthy individuals with severe eyesight impairment in the context of Charles Bonnet Syndrome (CBS) (80). Charles Bonnet Syndrome hallucinatory episodes can initially present to the sleep physician as reductions in visual input are exaggerated in the dark, causing events to occur primarily at night (98).

### Clinical and Polygraphic Features

Hypnagogic hallucinations are typical of the sleep-wake transition as demonstrated by the very few and limited studies (99), and may not be fully distinguishable from sleep onset dreams (77). Visual, auditory, and tactile sensations are the most reported (alone or co-occurring) (77). Visual phenomena include geometric patterns, shapes, and light flashes up to more complex kaleidoscopically changing and entoptic phenomena (100). Images involving faces, peoples and animals, and described as reality-like are possible and can be described as “highly detailed and colorful.” Voices and other sounds (phone, doorbell, and music) occur less commonly (83). They comprise vivid auditory impressions of words or names, people talking, but also environmental or animal sounds (101). Somatic experiences such as feelings of weightlessness, flying or falling, but also bodily distortions, and more rarely, a sense of presence in the room can occur (102). More rarely and frequently associated with underlying disorders or conditions, such as narcolepsy or altered sleep-wake cycle (92, 103), hypnagogic hallucinations can arise from sleep onset REM periods, sharing more similarities with hypnopompic hallucinations, which arise from a mixed state of REM and wake EEG (90). Dream ideation of REM sleep intruding into wakefulness gives hypnopompic hallucinations a greater emotional load, as unpleasant and frightening experiences, especially when associated with sleep paralysis, where the muscular atonia at EMG extends into wakefulness (85, 104). During these episodes, the person feels awake but unable to move, perceives ominous sounds (such as approaching footsteps), feels movement in the bed, and then feels (and/or smells) a person, creature or unspecified entity climbing upon the chest, a smothering sensation, and sometimes even a physical or sexual assault (102). In contrast to simple hypnagogic experiences, these events are typically accepted as vividly real, sometimes taken as assaults by human intruders but often interpreted as occult or metaphysical events (105). Sleep paralysis is also often accompanied by feelings of suffocation, as during these episodes auxiliary respiratory muscles (e.g., sternocleidomastoid and intercostals) are also paralyzed, but the diaphragm is not affected (104).

Finally, in the very few recorded sleep-related complex visual hallucinations, not related to any pre-disposing condition such as DLB or CBS, alpha rhythm, arising out of stages 2 and 3 NREM sleep was documented (81).

### Differential Diagnosis

The clinical picture of sleep related hallucinations clearly differentiates itself from Oneiric Stupor. In OS the patient is the protagonist of a single oneiric scene without any emotional involvement (22), in contrast with the passively experienced visual (or auditory) and emotionally loaded imaginary of hallucinations (77). The oneiric scene of OS is a “full screen” scenario not related to the nearby environment, while hallucinations are set against an existing background as one part of a perceived scene (94). Moreover, from the polygraphic point of view, hallucinations have been recorded as N1 or REM sleep onset periods from wake in an otherwise normal sleep/wake cycling (80, 103), while OS arises from a disrupted sleep structure (12).

### Dreams vs. Hallucinations: A Not So Easy Differential Physiopathology

On the other side, the true origin of these episodes has not been clearly discovered yet (80). Are sleep related hallucinations more similar to intruded dreams or limited hallucinations (as the ones seen in dementia, but also psychiatric disorders and drug abusers)? Phenomenological and physiopathological differences and similarities between dreams and hallucinations do occur (80, 94, 106). Neuroscientific and psychological theories tend to agree on the presence of overlapping mechanisms rather than distinct pathways as in the “gradual descent hypothesis” (107, 108). Neuroanatomical and neurophysiological evidence shows that mental functions during sleep involve a reorganization of the same systems which function during the day (1). Oscillatory neural activation and regional flow of information change progressively across the different sleep phases (108, 109) and EEG frequency and amplitude vary according to sleep depth. In a conceptual accordance, descriptions of mental events appear to follow a similar gradual continuum, starting with waking thoughts, hypnagogic hallucinations (at sleep onset), dreams, and finally hypnopompic hallucinations (with the re-emergence of wakefulness). Hence, from light sleep to NREM and REM dream periods, internal representations gradually evolve, starting with visual images only, subsequently adding somatic and auditory perceptions, followed by emotional contents and a narrative structure (80, 84, 110).

### Therapeutic Principles

When hypnagogic and hypnopompic hallucinations are isolated, patients need to be assured that they consist in a very frequent and (para)physiological phenomenon that occurs in healthy people and they are not a sign of psychosis, or a paranormal experience (111). When hallucinations become frequent or bothersome, pharmacological treatment with clomipramine 10–75 mg at bedtime may be effective, particularly when associated with sleep paralysis (112). Isolated sleep paralysis as well as hallucinations also benefit from adopting and following a correct sleep hygiene (113), as fragmented and/or disrupted sleep are frequently proximal causes (112). The same principles can be followed for complex visual hallucinations in their isolated form (111). When these conditions are secondary to other disorders such as narcolepsy or neurodegenerative disease, specific therapeutic options should be taken into consideration (92, 114).

## Nightmares and Nightmare Disorder

### Definition

Nightmares are common REM sleep-related parasomnias consisting in a frightening dream whose precise details are recalled on awakening. The well-remembered dysphoric dreams usually involve threats to survival, security, or physical integrity. Nightmares are worldwide experiences occasionally appearing during lifetime usually without any consequences (115). Recurrent nightmares causing clinically significant distress and/or impairment in social, occupational, or other important areas of functioning represent a nightmare disorder (116). According to the ICSD-3 the diagnosis of nightmare disorder consists of all the following criteria: (a) repeated occurrence of extended, extremely dysphoric, and well-remembered dreams that usually involve threats to survival, security, or physical integrity; (b) on awakening from the dysphoric dreams, the person rapidly becomes oriented and alert; (c) the dream experience, or the sleep disturbance produced by awakening from it, causes clinically significant distress or impairment in social, occupational, or other important areas of functioning (42).

### Associated Pathological Conditions

Nightmares can occur as a primary complaint or in the context of other sleep (e.g., insomnia and narcolepsy), psychiatric (stress, anxiety, depression, and schizophrenia), neurological (e.g., epilepsy), or systemic diseases or medication/substances intake (115–117).

Nightmares are a well-known symptom of post-traumatic stress disorder (PTSD), often beginning within 3 months of a trauma and sometimes persisting throughout life (118, 119). Nightmare disorder can cause sleep avoidance and phobia leading to sleep deprivation which is a possible factor of even more intense nightmares (115). Finally, a circadian rhythm misalignment and sleep deprivation caused by shift schedules could be implicated in nightmares, as they are more commonly reported by nurses working rotational shift work schedules compared to nurses working daytime only (120).

Recurrent nightmares may also be associated with the intake of various drugs, including antidepressants, antihypertensives (beta blockers, α-adrenergic receptor agonists, ACE inhibitors, sartans, and calcium antagonists), dopamine receptor agonists, cholinesterase blockers nicotinic acetylcholine blocker), and ganciclovir. Also, the abrupt withdrawal of REM sleep–suppressive agents (antidepressants, benzodiazepines, barbiturate, and ethanol) can induce nightmares (115).

### Epidemiology

Occasional nightmares are frequent, common both in childhood and in adulthood, occurring in an estimated 6.6% of the general population (85, 121). Even though nightmares often beginning during childhood could be stable over time, longitudinal studies, especially in adults are scarce (122).

### Clinical Features for Diagnosis

Little is known about the content of idiopathic nightmares. In a large prospective study, nightmares are more strongly related to themes involving a direct threat to physical integrity in comparison to bad dreams which present a broad range of thematic contents (123). The prevalent emotion in nightmares is fear and patients describe nightmares as substantially more emotionally intense than bad dreams. In addition, a significant proportion of bad dreams contains primary emotions other than fear, such as anger, sadness and frustration (124). Nightmares are significantly more bizarre than bad dreams which, in turn, are significantly more bizarre than everyday dreams (123).

Although there is an open debate about whether the dreamer needs to awaken from a dysphoric dream for the dream to be considered a nightmare (123), according to the American Academy of Sleep Medicine nightmares are defined as “*disturbing mental experiences rather than frightening dreams*” (42). Nightmares could constitute a sleep disorder or an independent mental disorder or a co-occurring disorder with another psychiatric condition (125). Daytime consequences such as tiredness upon getting up, daytime sleepiness, lack of energy and difficulties in concentrating could be present. Given their high prevalence and associated features of impaired mental and physical health, nightmares are of substantial clinical relevance, but studies documented that more than 60% of nightmare sufferers had never discussed nightmares with a clinician (126–128). Improving the identification of nightmare disorder, recommending that questions about nightmares should be included in sleep history taking and that patients should be offered an effective treatment is a goal to be achieved (116, 126).

### Polysomnographic Features

Nightmares generally occur in the last half of the night when REM sleep predominates. Overnight PSG is not routinely indicated to assess nightmare disorder but may be necessary to exclude other sleep behaviors and/or breathing disorders (115). Polygraphic recordings during nightmares occasionally show abrupt awakenings from REM sleep preceded by marked autonomic activation characterized by accelerated heart and respiratory rates. Of note, post-traumatic nightmares emerge both from REM and NREM sleep, including sleep onset (129).

### Physiopathology

The disposition-stress model hypothesizes that both nightmare frequency and neuroticism are significantly related to nightmare distress (130). Other research groups have underlined that the etiology of nightmare disorder may be influenced by increased hyperarousal that accumulates during the day and is maintained at night associated with an impaired fear extinction. Unlike what happens during normal sleep and dreaming, during which a process recombining fearful memories with novel and dissociated contexts produces fear extinction, in individuals with nightmare disorder arousing memory fragments during sleep, reinforcing fear memories are continuously activated (116). Several factors including mental and physical traumas and childhood adversity, trait susceptibility, maladaptive cognitive factors, and physiological factors have been proposed as facilitating hyperarousal and impaired fear extinction in patients diagnosed with nightmare disorder (116).

### Differential Diagnosis

Nightmares and nightmare disorder need to be distinguished from bad dreams, RBD, hypnagogic hallucinations with or without sleep paralysis, sleep terrors, and nocturnal panic attacks. Nightmares are often distinguished from bad dreams because the nightmares trigger an awakening whereas anxiety dreams are frightening dream experiences remembered only after waking up in the morning (123). Differently from RBD, during nightmares patients may vocalize or move minimally but complex motor behaviors with enacted dreams of defense against aggressions paralleling dream content usually are lacking (131). In addition, patients with nightmare disorder exhibit physiological REM atonia during polysomnography (85). Sleep terrors emerge from stage 3 NREM sleep with shouting and high autonomic changes. In contrast to sleep terror, in nightmares no confusion or disorientation is present, and the highly disturbing dream content frequently contrasts strikingly with relatively minor autonomic activation (except for the increase of autonomic tone before the awakenings) and no acting out of the nightmare (24, 132). Hallucinations and sleep paralysis may be described as “nightmares,” but they specifically occur at sleep onset and offset, and the paralysis affects the whole body (115). Nocturnal panic attacks are not associated with a detailed dreaming mentation. Severe sleep apnea may be associated with unpleasant sleep associated perceptions or images that resolve with the treatment of apnea (115).

### Therapeutic Principles

The best-established treatment of idiopathic nightmare disorder is image rehearsal therapy; systematic desensitization and progressive deep muscle relaxation training are suggested (116). Prazosin is recommended in PTSD-associated nightmares (133).

## Other Conditions

### Dream Mentation Is Not Exclusive of REM Sleep: The Example of Disorders of Arousal

Mental activity during the different stages of sleep varies regarding content and phenomenological characteristics [(134); Figure 1C]. Complex mentation is reported in 5–74% of non-rapid eye movement (NREM) sleep awakenings (135) in NREM parasomnias patients. Studying 45 adult DoA patients we found that 35 patients (77%) reported that, even if not constantly, they could recall some kind of mental activity at the end of the episode. Of them, 29 (64%) reported frightening and distressing contents, variably alternating with neutral contents in four of them. Fearful contents included someone chasing or trying to kill the patient, the ceiling falling on the patient, a truck running over the patient, mice infesting the house, being inside a box from which it was impossible to escape, a fire, walls crashing during an earthquake, thieves entering the house or a fox in the room (136, 137).

Classically mentation in REM is defined as “dreamlike,” defined by high emotional load, bizarre content and vivid images (138, 139). On the other hand, mentation during NREM sleep is addressed as “thought-like” and is usually less vivid and typically short. However, this phenomenological dichotomy was later demonstrated to be inconsistent as a significant amount of dreams reported from NREM sleep demonstrated typical aspects of “dreamlike” mentation (140, 141). Moreover, the control for length of the reports (e.g., word count), showed relatively similar qualitative features of REM and NREM dreams (138). Again, the “gradual descent hypothesis” (107, 108) reconciled the observed differences between mental experience during REM and NREM sleep stages as a result of continuous brain activity gradually differentiating from waking: REM and NREM mentation can be explained by stage-dependent physiological conditions of the brain (142).

### Lucid Dreaming

#### Definition

Lucid dreaming (LD) is a kind of consciousness state during which the dreamer is aware of the fact that he or she is dreaming, without leaving the sleeping state (143). LD is a non-pathological variant of normal REM dreaming. During lucid dreams individuals are physiologically asleep while at the same time aware that they are dreaming, able to intentionally perform voluntary actions in the dream scenario, and in some cases remember their waking life (144). In that state, individuals may keep some reflective consciousness and sometimes have partial control over the content of their dreams (145).

The gold standard technique to identify lucid dreams is to objectively verify the presence of distinct volitional eye movement patterns as recorded in the electrooculogram (EOG) during polysomnography-verified sleep in the so called “eye signaling technique.” The most common version of this procedure asks participants to signal when they realize they are dreaming by rapidly looking in the dream all the way to the left then all the way to the right two times consecutively then back to center without pausing (146, 147).

#### Associated Pathological Conditions

Only one paper describes two young patients who reported frequent lucid dreams and increased nocturnal awakenings after unilateral ischemic stroke of the left mediodorsal thalamus (148). Narcoleptic patents experience a higher lucid dreaming frequency which has also been correlated to an increase in creativity in these patients (149).

#### Etiophysiology

Evidence suggests that lucid dreaming can be learnt by training in prospective memory techniques (143, 147), interrupting sleep with short periods of wakefulness (143, 150) or by means of eye-signaling methods (146).

Cholinergic enhancement with an acetylcholinesterase inhibitor such as galantamine seems to facilitate lucid dreams; recreational drugs such as alcohol, cocaine, and cannabis have been suggested to have the same effects by suppressing REM sleep (151, 152), leading to a subsequent REM rebound (153). Rapid eye movement rebound could potentially increase lucid dream in pre-disposed individuals. Finally, another example is LSD, as it could prolong REM sleep at certain doses (154), which could potentially be favorable to lucid dreaming.

#### Epidemiology

Most people spontaneously experience lucid dreams infrequently. Lucid dream frequency varies from never (~40–50%) to monthly (~20%) to a small percentage of people that report lucid dreams several times per week or in some cases every night (155–157).

#### Polysomnographic Features

Lucid dreams predominantly occur in REM sleep (147), in particular during activated periods of REM sleep, characterized by increased phasic activity (e.g., increased REM density) and higher autonomic nervous system arousal (e.g., heart rate, respiration rate, and skin potential) (158). LD were also rarely reported during sleep stages N1 and N2 or in an unambiguous stage of NREM sleep (150, 159–161). Different studies have observed an increase in central or posterior alpha, parietal beta, fronto-lateral gamma or a reduction in frontocentral delta during lucid dreams compared to baseline REM sleep, although EEG studies show substantial disagreement regarding the spatial and spectral changes associated with lucid dreaming (146).

## Conclusion

Oneiric stupor is a peculiar condition different from the other disorders of dreaming. It can be considered neither a dream nor a hallucination, indeed, from a phenomenological point of view, its features are the following. First, it consists in a single oneiric scene (in terms of both scenario and occurrence) rather than a structured “dream tale.” The scene takes place in a “full screen” scenario differently from the superimposed perception of hallucinations. The patient is the only protagonist of the oneiric scene, developing hand in hand with the motor behavior, characterized by stereotyped and repetitive gestures influenced by the patient's routine or hobbies. The occurrence involves little or no volition and reflective consciousness; emotional load is completely absent, probably as a secondary result of the complete disconnection from thalamo-limbic circuits (18). A complete derangement of sleep structure characterized by the alternation of wake, REM sleep, and subwakefulness determines the neurophysiological substrate of oneiric stupor (26).

Clinical and neurophysiological observation of this condition suggested general reflections on the composition and function of the cerebral neuronal network generating wake and sleep behavior (12, 18, 30). On the basis of the data on Agrypnia excitata and some experimental findings, Lugaresi, Montagna, and collaborators suggested that sleep originates from a widespread neuronal network extending from the reticular brainstem formation to the limbic cortex, where three distinct generators are involved following a caudorostral organization (30).

**Figure 3:**
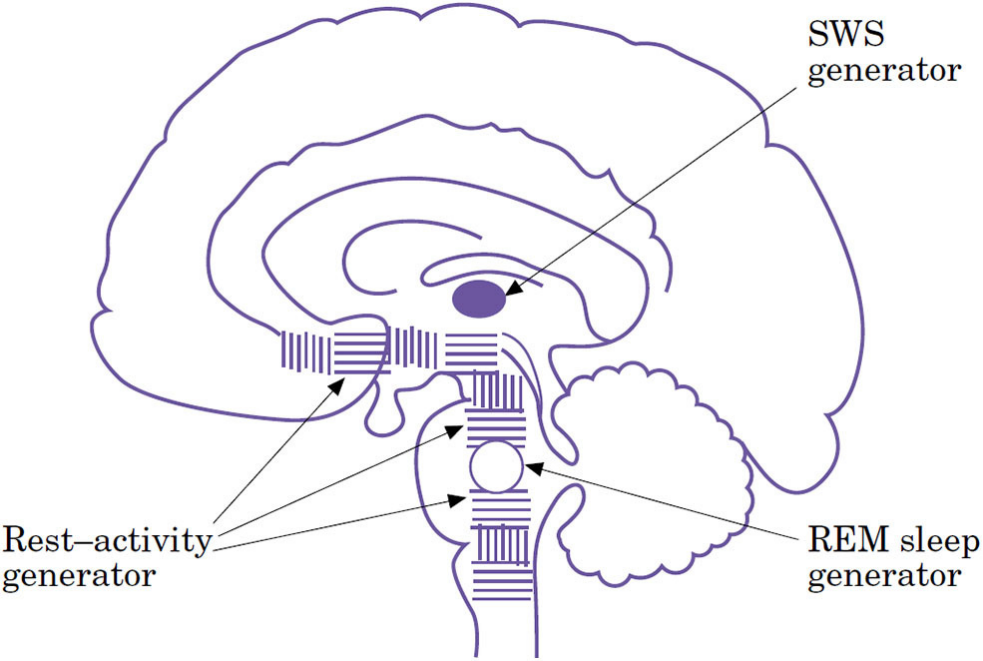
Schematic diagram of the neuronal structures responsible for sleep generation suggesting three different types of sleep and respective sleep generators [From (18), with permissions].

The first generator diffuses from the medulla to the basal forebrain within the so-called extended reticular formation, controls, and organizes the vigilance levels behaviorally expressed by three essential features: active wakefulness, quiet wakefulness, and pre-sleep behavior. Slow-wave sleep and REM sleep have two distinct and localized generators, respectively located at the telencephalic and rhombencephalic levels (Figure 3).

The caudalmost part of the network, as shown by experimental medullary and midpontine preparations (162) is still able to organize rest–activity cycles alone. At a more rostral level, the integrity of the rostral pons and the caudal midbrain is both necessary and sufficient to generate the basic aspects of REM sleep. In fact, three different behaviors can occur in the midbrain transected animal: wakefulness, REM sleep, and a third peculiar condition characterized by postural rest, slow eye movements, and fluctuating myosis mimicking NREM stage 1 of human sleep (25, 26, 163). However, only if the third most elevated level of functional integration, including the thalamus and thalamocortical loops, is operating spindles and delta sleep are activated (27).

According to this view, drowsiness/stage 1 NREM must be dissected from proper sleep stages (NREM stage 2 and 3, and REM). Oneiric stupor represents the phenomenological manifestation of removing slow-wave sleep from the equation.

## Author Contributions

LB curated the first draft of the work with substantial contributions to the sections Oneiric Stupor in Agrypnia excitata, REM Sleep Behavior Disorder, and Sleep Related Hallucinations. FP curated the sections Nightmares and Other Disorders and gave substantial contributions in performing the critical revision of the manuscript. All authors contributed to the article and approved the submitted version.

## Conflict of Interest

FP received honoraria for consultancies from Vanda Pharmaceutical, Sanofi and Zambon, and honoraria for lectures from Fidia, Bial, Eisai Japan, and Italfarmaco, all outside the submitted work. The remaining author declares that the research was conducted in the absence of any commercial or financial relationships that could be construed as a potential conflict of interest.

## References

[1] Nir Y Tononi G. Dreaming and the brain: from phenomenology to neurophysiology. Trends Cogn Sci. (2010) 14:88–100. 10.1016/j.tics.2009.12.001 20079677 PMC2814941

[2] Shaw B Developments in the Neuroscience of Dreams. Activitas Nervosa Superior (2016) 58:45–50.

[3] Mutz J Javadi AH. Exploring the neural correlates of dream phenomenology and altered states of consciousness during sleep. Neurosci Conscious. (2017) 2017:nix009. 10.1093/nc/nix009 30042842 PMC6007136

[4] Eiser AS. Physiology and psychology of dreams. Semin Neurol. (2005) 25:97–105. 10.1055/s-2005-867078 15798942

[5] Desseilles M Dang-Vu TT Sterpenich V Schwartz S. Cognitive and emotional processes during dreaming: a neuroimaging view. Conscious Cogn. (2011) 20:998–1008. 10.1016/j.concog.2010.10.005 21075010

[6] Hobson JA. REM sleep and dreaming: towards a theory of protoconsciousness. Nat Rev Neurosci. (2009) 10:803–13. 10.1038/nrn2716 19794431

[7] Horikawa T Tamaki M Miyawaki Y Kamitani Y. Neural decoding of visual imagery during sleep. Science. (2013) 340:639–42. 10.1126/science.1234330 23558170

[8] Cipolli C Ferrara M De Gennaro L Plazzi G. Beyond the neuropsychology of dreaming: Insights into the neural basis of dreaming with new techniques of sleep recording and analysis. Sleep Med Rev. (2017) 35:8–20. 10.1016/j.smrv.2016.07.005 27569701

[9] Siclari F Baird B Perogamvros L Bernardi G LaRocque JJ Riedner B. The neural correlates of dreaming. Nat Neurosci. (2017) 20:872–8. 10.1038/nn.4545 28394322 PMC5462120

[10] Montagna P. Chapter 59—Fatal familial insomnia and the role of the thalamus in sleep regulation. In: Montagna P Chokroverty S editors. Handbook of Clinical Neurology. Amsterdam: Elsevier (2011). p. 981–96. 10.1016/B978-0-444-52007-4.00018-7 21056239

[11] Genette G Narrative Discourse: An Essay in Method. Ithaca, NY: Cornell University Press (1980).

[12] Montagna P Lugaresi E. Agrypnia excitata: a generalized overactivity syndrome and a useful concept in the neurophysiopathology of sleep. Clin Neurophysiol. (2002) 113:552–60. 10.1016/S1388-2457(02)00022-6 11956000

[13] Provini F Cortelli P Montagna P Gambetti P Lugaresi E. Fatal insomnia and agrypnia excitata: sleep and the limbic system. Rev Neurol (Paris). (2008) 164:692–700. 10.1016/j.neurol.2007.11.003 18805303

[14] Kong Q Surewicz WK Petersen RB Zou W Chen SG Gambetti P Inherited prion diseases. In: Prusiner SB editor. Prion Biology and Diseases. 2nd. New York, NY: Cold Spring Harbor Laboratory Press (2004) 673–76.

[15] Montagna P Cortelli P Avoni P Tinuper P Plazzi G Gallassi R. Clinical features of fatal familial insomnia: phenotypic variability in relation to a polymorphism at codon 129 of the prion protein gene. Brain Pathol. (1998) 8:515–20. 10.1111/j.1750-3639.1998.tb00172.x 9669701 PMC8098256

[16] Liguori R Vincent A Clover L Avoni P Plazzi G Cortelli P. Morvan's syndrome: peripheral and central nervous system and cardiac involvement with antibodies to voltage-gated potassium channels. Brain. (2001) 124:2417–26. 10.1093/brain/124.12.2417 11701596

[17] Plazzi G Montagna P Meletti S Lugaresi E. Polysomnographic study of sleeplessness and oneiricisms in the alcohol withdrawal syndrome. Sleep Med. (2002) 3:279–82. 10.1016/S1389-9457(02)00014-X 14592220

[18] Lugaresi E Provini F. Agrypnia excitata: clinical features and pathophysiological implications. Sleep Med Rev. (2001) 5:313–22. 10.1053/smrv.2001.0166 12530995

[19] Calandra-Buonaura G Provini F Guaraldi P Pizza F Cecere A Barletta G. Oculomasticatory myorhythmia and agrypnia excitata guide the diagnosis of Whipple disease. Sleep Med. (2013) 14:1428–30. 10.1016/j.sleep.2013.06.022 24210608

[20] Ferri R Lanuzza B Cosentino FI Iero I Russo N Tripodi M. Agrypnia excitata in a patient with progeroid short stature and pigmented nevi (Mulvihill-Smith syndrome). J Sleep Res. (2005) 14:463–70. 10.1111/j.1365-2869.2005.00465.x 16364148

[21] La Morgia C Parchi P Capellari S Lodi R Tonon C Rinaldi R. ‘Agrypnia excitata’ in a case of sporadic Creutzfeldt-Jakob disease VV2. J Neurol Neurosurg Psychiatry. (2009) 80:244–6. 10.1136/jnnp.2008.149344 19151026

[22] Guaraldi P Calandra-Buonaura G Terlizzi R Montagna P Lugaresi E Tinuper P. Oneiric stupor: the peculiar behaviour of agrypnia excitata. Sleep Med. (2011) 12(Suppl. 2):S64–7. 10.1016/j.sleep.2011.10.014 22136903

[23] Baldelli L Provini F. Fatal familial insomnia and agrypnia excitata: autonomic dysfunctions and pathophysiological implications. Auton Neurosci Basic Clin. (2019) 218:68–86. 10.1016/j.autneu.2019.02.007 30890351

[24] Loddo G Lopez R Cilea R Dauvilliers Y Provini F Disorders of arousal in adults: new diagnostic tools for clinical practice. Sleep Sci Pract. (2019) 3:5 10.1186/s41606-019-0037-3

[25] Lugaresi E Provini F. Fatal familial insomnia and agrypnia excitata. Rev Neurol Dis. (2007) 4:145–52. 17943067

[26] Lugaresi E Provini F Cortelli P Agrypnia excitata. Sleep Med. (2011) 12(Suppl. 2):S3–10. 10.1016/j.sleep.2011.10.004 22136896

[27] Provini F. Agrypnia excitata. Curr Neurol Neurosci Rep. (2013) 13:341. 10.1007/s11910-013-0341-8 23423537

[28] Sforza E Montagna P Tinuper P Cortelli P Avoni P Ferrillo F. Sleep-wake cycle abnormalities in fatal familial insomnia. Evidence of the role of the thalamus in sleep regulation. Electroencephalogr Clin Neurophysiol. (1995) 94:398–405. 10.1016/0013-4694(94)00318-F 7607093

[29] Tinuper P Montagna P Medori R Cortelli P Zucconi M Baruzzi A. The thalamus participates in the regulation of the sleep-waking cycle. A clinico-pathological study in fatal familial thalamic degeneration. Electroencephalogr Clin Neurophysiol. (1989) 73:117–23. 10.1016/0013-4694(89)90190-9 2473878

[30] Lugaresi E Provini F Montagna P The neuroanatomy of sleep. Considerations on the role of the thalamus in sleep and a proposal for a caudorostral organization. Eur J Anat. (2004) 8:85–93.

[31] Montagna P. Fatal familial insomnia: a model disease in sleep physiopathology. Sleep Med Rev. (2005) 9:339–53. 10.1016/j.smrv.2005.02.001 16109494

[32] Steriade M McCormick DA Sejnowski TJ. Thalamocortical oscillations in the sleeping and aroused brain. Science. (1993) 262:679–85. 10.1126/science.8235588 8235588

[33] Jouvet M. Research on the neural structures and responsible mechanisms in different phases of physiological sleep. Arch Ital Biol. (1962) 100:125–206. 14452612

[34] Lu J Sherman D Devor M Saper CB. A putative flip-flop switch for control of REM sleep. Nature. (2006) 441:589–94. 10.1038/nature04767 16688184

[35] Saper CB Fuller PM Pedersen NP Lu J Scammell TE. Sleep state switching. Neuron. (2010) 68:1023–42. 10.1016/j.neuron.2010.11.032 21172606 PMC3026325

[36] Cracco L Appleby BS Gambetti P. Fatal familial insomnia and sporadic fatal insomnia. Handb Clin Neurol. (2018) 153:271–99. 10.1016/B978-0-444-63945-5.00015-5 29887141

[37] Will RG Campbell MJ Moss TH Bell JE Ironside JW. FFI cases from the United Kingdom. Brain Pathol. (1998) 8:562–3. 10.1111/j.1750-3639.1998.tb00182.x 9669711 PMC8098161

[38] Montagna P Cortelli P Tinuper P Fatal familial insomnia: a disease that emphasizes the role of the thalamus in the regulation of sleep and vegetative functions. In: Guilleminault C Lugaresi E Montagna P Gambetti P editors. Fatal Familial Insomnia: Inherited Prion Diseases, Sleep, and the Thalamus. New York, NY: Raven Press (1994). p. 1–14.

[39] Fischer-Perroudon C Trillet M Mouret J Tommasi M Jouvet M Schott B. Polygraphic and metabolic studies of persistent insomnia with hallucinations. Apropos of an antomo-clinical study of a case of Morvan's fibrillar chorea. Rev Neurol (Paris). (1974) 130:111–25. 4548556

[40] Masood W Sitammagari KK. Morvan Syndrome (Morvan Fibrillary Chorea, MFC). Treasure Island, FL: StatPearls. (2020).

[41] Josephs KA Silber MH Fealey RD Nippoldt TB Auger RG Vernino S. Neurophysiologic studies in Morvan syndrome. J Clin Neurophysiol. (2004) 21:440–5. 10.1097/00004691-200411000-00008 15622131

[42] American Academy of Sleep Medicine International Classification of Sleep Disorders: Diagnostic and Coding Manual. 3rd ed. Darien, IL: American Academy of Sleep Medicine (2014).

[43] Sixel-Doring F Zimmermann J Wegener A Mollenhauer B Trenkwalder C. The evolution of REM sleep behavior disorder in early Parkinson disease. Sleep. (2016) 39:1737–42. 10.5665/sleep.6102 27306265 PMC4989262

[44] Dugger BN Boeve BF Murray ME Parisi JE Fujishiro H Dickson DW. Rapid eye movement sleep behavior disorder and subtypes in autopsy-confirmed dementia with Lewy bodies. Mov Disord. (2012) 27:72–8. 10.1002/mds.24003 22038951 PMC3513369

[45] McKeith IG Boeve BF Dickson DW Halliday G Taylor JP Weintraub D. Diagnosis and management of dementia with Lewy bodies: fourth consensus report of the DLB Consortium. Neurology. (2017) 89:88–100. 10.1212/WNL.0000000000004058 28592453 PMC5496518

[46] Palma JA Fernandez-Cordon C Coon EA Low PA Miglis MG Jaradeh S. Prevalence of REM sleep behavior disorder in multiple system atrophy: a multicenter study and meta-analysis. Clin Auton Res. (2015) 25:69–75. 10.1007/s10286-015-0279-9 25739474 PMC4406814

[47] Silber MH Autoimmune sleep disorders. Handb Clin Neurol. (2016) 133:317–26. 10.1016/B978-0-444-63432-0.00018-9 27112685

[48] McCarter SJ Tippmann-Peikert M Sandness DJ Flanagan EP Kantarci K Boeve BF. Neuroimaging-evident lesional pathology associated with REM sleep behavior disorder. Sleep Med. (2015) 16:1502–10. 10.1016/j.sleep.2015.07.018 26611948

[49] Provini F Tachibana N. Acute REM sleep behavior disorder. In: Schenck C, Högl B, Videnovic A, editors. Rapid-Eye-Movement Sleep Behavior Disorder. Cham: Springer (2019). p. 153–71. Available online at: http://doi-org-443.webvpn.fjmu.edu.cn/10.1007/978-3-319-90152-7_12

[50] Dauvilliers Y Schenck CH Postuma RB Iranzo A Luppi PH Plazzi G. REM sleep behaviour disorder. Nat Rev Dis Primers. (2018) 4:19. 10.1038/s41572-018-0016-5 30166532

[51] Postuma RB Iranzo A Hu M Hogl B Boeve BF Manni R. Risk and predictors of dementia and parkinsonism in idiopathic REM sleep behaviour disorder: a multicentre study. Brain. (2019) 142:744–59. 10.1093/brain/awz030 30789229 PMC6391615

[52] Schenck CH Boeve BF Mahowald MW. Delayed emergence of a parkinsonian disorder or dementia in 81% of older men initially diagnosed with idiopathic rapid eye movement sleep behavior disorder: a 16-year update on a previously reported series. Sleep Med. (2013) 14:744–8. 10.1016/j.sleep.2012.10.009 23347909

[53] Iranzo A Santamaria J Tolosa E. Idiopathic rapid eye movement sleep behaviour disorder: diagnosis, management, and the need for neuroprotective interventions. Lancet Neurol. (2016) 15:405–19. 10.1016/S1474-4422(16)00057-0 26971662

[54] Iranzo A Stefani A Serradell M Marti MJ Lomena F Mahlknecht P. Characterization of patients with longstanding idiopathic REM sleep behavior disorder. Neurology. (2017) 89:242–8. 10.1212/WNL.0000000000004121 28615430

[55] Högl B Stefani A Videnovic A. Idiopathic REM sleep behaviour disorder and neurodegeneration—an update. Nat Rev Neurol. (2018) 14:40–55. 10.1038/nrneurol.2017.157 29170501

[56] Kang SH Yoon IY Lee SD Han JW Kim TH Kim KW. REM sleep behavior disorder in the Korean elderly population: prevalence and clinical characteristics. Sleep. (2013) 36:1147–52. 10.5665/sleep.2874 23904674 PMC3700711

[57] Haba-Rubio J Frauscher B Marques-Vidal P Toriel J Tobback N Andries D. Prevalence and determinants of rapid eye movement sleep behavior disorder in the general population. Sleep. (2018) 41:zsx197. 10.1093/sleep/zsx197 29216391

[58] Fernandez-Arcos A Iranzo A Serradell M Gaig C Santamaria J. The clinical phenotype of idiopathic rapid eye movement sleep behavior disorder at presentation: a study in 203 consecutive patients. Sleep. (2016) 39:121–32. 10.5665/sleep.5332 26940460 PMC4678361

[59] Pérez-Carbonell L Iranzo A Clinical aspects of idiopathic RBD. In: Schenck CH Högl B Videnovic A editors. Rapid-Eye-Movement Sleep Behavior Disorder. Cham: Springer International Publishing (2019). p. 33–52.

[60] Frauscher B Gschliesser V Brandauer E Marti I Furtner MT Ulmer H. REM sleep behavior disorder in 703 sleep-disorder patients: the importance of eliciting a comprehensive sleep history. Sleep Med. (2010) 11:167–71. 10.1016/j.sleep.2009.03.011 20022299

[61] Ramos-Campoy O Gaig C Villas M Iranzo A Santamaria J. REM sleep behavior disorder causing subdural hematoma. Sleep Med. (2017) 30:43–4. 10.1016/j.sleep.2016.09.025 28215261

[62] Arnulf I. RBD: a window into the dreaming process. In: Schenck CH Högl B Videnovic A editors. Rapid-Eye-Movement Sleep Behavior Disorder. Cham: Springer International Publishing (2019). p. 223–42.

[63] Oudiette D Leu-Semenescu S Roze E Vidailhet M De Cock VC Golmard JL. A motor signature of REM sleep behavior disorder. Mov Disord. (2012) 27:428–31. 10.1002/mds.24044 22173891

[64] Herlin B Leu-Semenescu S Chaumereuil C Arnulf I. Evidence that non-dreamers do dream: a REM sleep behaviour disorder model. J Sleep Res. (2015) 24:602–9. 10.1111/jsr.12323 26307463

[65] Berry R Brooks R Gamaldo C Harding S Lloyd R Marcus C. The AASM Manual for the Scoring of Sleep and Associated Events: Rules, Terminology and Technical Specifications. Version 2.6. Darien, IL: American Academy of Sleep Medicine (2020).

[66] Zhang J Lam SP Ho CK Li AM Tsoh J Mok V. Diagnosis of REM sleep behavior disorder by video-polysomnographic study: is one night enough? Sleep. (2008) 31:1179–85. 10.5665/sleep/31.8.1179 18714790 PMC2542964

[67] Stefani A Frauscher B Högl B Diagnosis of REM sleep behavior disorder. In: Schenck CH Högl B Videnovic A editors. Rapid-Eye-Movement Sleep Behavior Disorder. Cham: Springer International Publishing (2019). p. 245–54.

[68] Puligheddu M Congiu P Ferri R. The electromyographic diagnosis of REM sleep without atonia and REM sleep behavior disorder. In: Schenck CH Högl B Videnovic A editors. Rapid-Eye-Movement Sleep Behavior Disorder. Cham: Springer International Publishing (2019). p. 447–64.

[69] Montplaisir J Gagnon JF Fantini ML Postuma RB Dauvilliers Y Desautels A Polysomnographic diagnosis of idiopathic REM sleep behavior disorder. Mov Disord. (2010) 25:2044–51. 10.1002/mds.23257 20818653

[70] Fantini ML Michaud M Gosselin N Lavigne G Montplaisir J. Periodic leg movements in REM sleep behavior disorder and related autonomic and EEG activation. Neurology. (2002) 59:1889–94. 10.1212/01.WNL.0000038348.94399.F6 12499479

[71] Luppi P-H Clément O Sapin E Gervasoni D Peyron C Léger L. The neuronal network responsible for paradoxical sleep and its dysfunctions causing narcolepsy and rapid eye movement (REM) behavior disorder. Sleep Med Rev. (2011) 15:153–63. 10.1016/j.smrv.2010.08.002 21115377

[72] Boeve BF Silber MH Saper CB Ferman TJ Dickson DW Parisi JE. Pathophysiology of REM sleep behaviour disorder and relevance to neurodegenerative disease. Brain. (2007) 130:2770–88. 10.1093/brain/awm056 17412731

[73] Mahowald MW Schenck CH. The REM sleep behavior disorder odyssey. Sleep Med Rev. (2009) 13:381–4. 10.1016/j.smrv.2009.02.002 19394252

[74] Blumberg MS Plumeau AM. A new view of “dream enactment” in REM sleep behavior disorder. Sleep Med Rev. (2016) 30:34–42. 10.1016/j.smrv.2015.12.002 26802823 PMC4912466

[75] Tiriac A Del Rio-Bermudez C Blumberg Mark S. Self-generated movements with “Unexpected” sensory consequences. Curr Biol. (2014) 24:2136–41. 10.1016/j.cub.2014.07.053 25131675 PMC4175005

[76] Provini F Marconi S Amadori M Guaraldi P Pierangeli G Cortelli P. Morvan chorea and agrypnia excitata: when video-polysomnographic recording guides the diagnosis. Sleep Med. (2011) 12:1041–3. 10.1016/j.sleep.2011.05.005 22033118

[77] Pagel JF Pandi-Perumal SR Dreaming and sleep disorder. In: Chokroverty S, editor. Sleep Disorders Medicine. New York, NY: Springer (2017) p. 225–34. Available online at: http://doi-org-443.webvpn.fjmu.edu.cn/10.1007/978-1-4939-6578-6_14

[78] Howell MJ Management of a patient with RBD. In: Schenck CH Högl B Videnovic A editors. Rapid-Eye-Movement Sleep Behavior Disorder. Cham: Springer International Publishing (2019). p. 305–14.

[79] Aurora RN Zak RS Maganti RK Auerbach SH Casey KR Chowdhuri S. Best practice guide for the treatment of REM sleep behavior disorder (RBD). J Clin Sleep Med. (2010) 6:85–95. 10.5664/jcsm.27717 20191945 PMC2823283

[80] Waters F Blom JD Dang-Vu TT Cheyne AJ Alderson-Day B Woodruff P. What is the link between hallucinations, dreams, and hypnagogic-hypnopompic experiences? Schizophr Bull. (2016) 42:1098–109. 10.1093/schbul/sbw076 27358492 PMC4988750

[81] Silber MH Hansen MR Girish M. Complex nocturnal visual hallucinations. Sleep Med. (2005) 6:363–6. 10.1016/j.sleep.2005.03.002 15946898

[82] Manford M Andermann F. Complex visual hallucinations. Clinical and neurobiological insights. Brain. (1998) 121:1819–40. 10.1093/brain/121.10.1819 9798740

[83] Ohayon MM Priest RG Caulet M Guilleminault C. Hypnagogic and hypnopompic hallucinations: pathological phenomena? Br J Psychiatry. (1996) 169:459–67. 10.1192/bjp.169.4.459 8894197

[84] Foulkes D Vogel G. Mental activity at sleep onset. J Abnorm Psychol. (1965) 70:231–43. 10.1037/h0022217 14341704

[85] Stefani A Holzknecht E Hogl B. Clinical neurophysiology of REM parasomnias. Handb Clin Neurol. (2019) 161:381–96. 10.1016/B978-0-444-64142-7.00062-X 31307615

[86] Chase MH The control of motoneurons during sleep. In: Kryger MH Roth T Dement WC editors. Principles and Practice of Sleep Medicine. 2nd ed. Philadelphia, PA: Saunders (1994). p. 163–75.

[87] Siegel JM Chapter 8—Rapid eye movement sleep. In: Kryger M Roth T Dement WC editors. Principles and Practice of Sleep Medicine. 6th ed. Philadelphia, PA: Elsevier (2017). p. 78–95.e6.

[88] Hishikawa Y Shimizu T. Physiology of REM sleep, cataplexy, and sleep paralysis. Adv Neurol. (1995) 67:245–71. 8848973

[89] McCarty DE Chesson AL. A case of sleep paralysis with hypnopompic hallucinations. J Clin Sleep Med. (2009) 5:83–4. 10.5664/jcsm.27398 19317387 PMC2637172

[90] Hogl B Iranzo A. Rapid eye movement sleep behavior disorder and other rapid eye movement sleep parasomnias. Continuum. (2017) 23:1017–34. 10.1212/CON.0000000000000489 28777174

[91] Frauscher B Ehrmann L Mitterling T Gabelia D Gschliesser V Brandauer E. Delayed diagnosis, range of severity, and multiple sleep comorbidities: a clinical and polysomnographic analysis of 100 patients of the innsbruck narcolepsy cohort. J Clin Sleep Med. (2013) 9:805–12. 10.5664/jcsm.2926 23946711 PMC3716672

[92] Bassetti CLA Adamantidis A Burdakov D Han F Gay S Kallweit U. Narcolepsy—clinical spectrum, aetiopathophysiology, diagnosis and treatment. Nat Rev Neurol. (2019) 15:519–39. 10.1038/s41582-019-0226-9 31324898

[93] Wamsley E Donjacour CE Scammell TE Lammers GJ Stickgold R. Delusional confusion of dreaming and reality in narcolepsy. Sleep. (2014) 37:419–22. 10.5665/sleep.3428 24501437 PMC3900627

[94] Collerton D Perry E. Dreaming and hallucinations—continuity or discontinuity? Perspectives from dementia with Lewy bodies. Conscious Cogn. (2011) 20:1016–20. 10.1016/j.concog.2011.03.024 21531149

[95] Esmaeeli S Murphy K Swords GM Ibrahim BA Brown JW Llano DA. Visual hallucinations, thalamocortical physiology and lewy body disease: a review. Neurosci Biobehav Rev. (2019) 103:337–51. 10.1016/j.neubiorev.2019.06.006 31195000

[96] Mosimann UP Rowan EN Partington CE Collerton D Littlewood E O'Brien JT. Characteristics of visual hallucinations in Parkinson disease dementia and dementia with lewy bodies. Am J Geriatr Psychiatry. (2006) 14:153–60. 10.1097/01.JGP.0000192480.89813.80 16473980

[97] Lai S Bruce V Collerton D Visual hallucinations in older people: appraisals but not content or phenomenology influence distress. Behav Cogn Psychother. (2016) 44:705–10. 10.1017/S1352465815000727 26530689

[98] Lipford MC Sandness DJ St Louis EK. A 69-year-old man with complex nocturnal visual hallucinations. J Clin Sleep Med. (2015) 11:491–3. 10.5664/jcsm.4614 25665691 PMC4365464

[99] Hori T Hayashi M Morikawa T Topographical EEG changes and the hypnagogic experience. In: Ogilvie RD Harsh JP editors. Sleep Onset: Normal and Abnormal Processes. Washington, DC: American Psychological Association (1994). p. 237–53.

[100] Leroy EB Les Visions du Demi-Sommeil: Hallucinations Hypnagogiques. Paris: Librairie Félix Alcan (1933).

[101] Jones SR Fernyhough C Larøi F A phenomenological survey of auditory verbal hallucinations in the hypnagogic and hypnopompic states. Phenomenol Cogn Sci. (2010) 9:213–24. 10.1007/s11097-010-9158-y

[102] Cheyne JA Rueffer SD Newby-Clark IR. Hypnagogic and hypnopompic hallucinations during sleep paralysis: neurological and cultural construction of the night-mare. Conscious Cogn. (1999) 8:319–37. 10.1006/ccog.1999.0404 10487786

[103] Takeuchi T Miyasita A Inugami M Sasaki Y Fukuda K. Laboratory-documented hallucination during sleep-onset REM period in a normal subject. Percept Mot Skills. (1994) 78:979–85. 10.1177/003151259407800355 8084722

[104] Takeuchi T Miyasita A Sasaki Y Inugami M Fukuda K. Isolated sleep paralysis elicited by sleep interruption. Sleep. (1992) 15:217–25. 10.1093/sleep/15.3.217 1621022

[105] Cheyne JA Pennycook G Sleep paralysis postepisode distress: modeling potential effects of episode characteristics, general psychological distress, beliefs, and cognitive style. Clin Psychol Sci. (2013) 1:135–48. 10.1177/2167702612466656

[106] Jalal B The neuropharmacology of sleep paralysis hallucinations: serotonin 2A activation and a novel therapeutic drug. Psychopharmacology (Berl). (2018) 235:3083–91. 10.1007/s00213-018-5042-1 30288594 PMC6208952

[107] Fosse R Stickgold R Hobson JA. The mind in REM sleep: reports of emotional experience. Sleep. (2001) 24:947–55. 10.1093/sleep/24.8.1 11766165

[108] Dinges DF Are you awake? Cognitive performance and reverie during the hypnopompic state. In: Bootzin RR Kihlstrom JF Schacter DL editors. Sleep and Cognition. Washington, DC: American Psychological Association (1990). p. 159–75.

[109] Carskadon MA Dement WC Chapter 2—Normal human sleep: an overview. In: Kryger M Roth T Dement WC editors. Principles and Practice of Sleep Medicine. 6th ed. Philadelphia, PA: Elsevier (2017). p. 15–24.e3.

[110] Rowley JT Stickgold R Hobson JA. Eyelid movements and mental activity at sleep onset. Conscious Cogn. (1998) 7:67–84. 10.1006/ccog.1998.0333 9521833

[111] Iranzo A Chapter 105—Other parasomnias. In: Kryger M Roth T Dement WC editors. Principles and Practice of Sleep Medicine. 6th ed. Philadelphia, PA: Elsevier (2017). p. 1011–9.e4.

[112] Sharpless BA A clinician's guide to recurrent isolated sleep paralysis. Neuropsychiatr Dis Treat. (2016) 12:1761–7. 10.2147/NDT.S100307 27486325 PMC4958367

[113] Jack DE Colleen EC Overcoming Insomnia A Cognitive-Behavioral Therapy Approach, Therapist Guide. New York, NY: Oxford University Press (2015).

[114] Bonnet U Taha S Stuehler L Knierim U. The transition from vivid dreams over floccillations and visual hallucinations to complete delirium in a geriatric patient at the dawn of Alzheimer's dementia: beneficial role of rivastigmine. Psychogeriatrics. (2019) 19:404–6. 10.1111/psyg.12412 30723981

[115] Arnulf I Chapter 104—nightmares and dream disturbances. In: Kryger M Roth T Dement WC editors. Principles and Practice of Sleep Medicine. 6th ed. Philadelphia, PA: Elsevier (2017). p. 1002–10.e4.

[116] Gieselmann A Ait Aoudia M Carr M Germain A Gorzka R Holzinger B. Aetiology and treatment of nightmare disorder: state of the art and future perspectives. J Sleep Res. (2019) 28:e12820. 10.1111/jsr.12820 30697860 PMC6850667

[117] Baldelli L Addimanda O Burattini M Chiaro G Brusi V Pignotti E. Nightmare disorder and REM sleep behavior disorder in inflammatory arthritis: possibility beyond neurodegeneration. Brain Behav. (2019) 9:e01230. 10.1002/brb3.1230 30770647 PMC6422707

[118] Trauma- and Stressor-Related Disorders In: American Psychiatric Association. Diagnostic and Statistical Manual of Mental Disorders. 5th ed. Washington, DC: American Psychiatric Association Press (2013).

[119] Ohayon MM Shapiro CM. Sleep disturbances and psychiatric disorders associated with posttraumatic stress disorder in the general population. Compr Psychiatry. (2000) 41:469–78. 10.1053/comp.2000.16568 11086154

[120] Bjorvatn B Mageroy N Moen BE Pallesen S Waage S Parasomnias are more frequent in shift workers than in day workers. Chronobiol Int. (2015) 32:1352–8. 10.3109/07420528.2015.1091354 26540469

[121] Ohayon MM Schenck CH Violent behavior during sleep: prevalence, comorbidity and consequences. Sleep Med. (2010) 11:941–6. 10.1016/j.sleep.2010.02.016 20817553 PMC2939252

[122] Schredl M Nightmare frequency and nightmare topics in a representative German sample. Eur Arch Psychiatry Clin Neurosci. (2010) 260:565–70. 10.1007/s00406-010-0112-3 20229263

[123] Robert G Zadra A. Thematic and content analysis of idiopathic nightmares and bad dreams. Sleep. (2014) 37:409–17. 10.5665/sleep.3426 24497669 PMC3900621

[124] Sandman N Valli K Kronholm E Ollila HM Revonsuo A Laatikainen T. Nightmares: prevalence among the Finnish general adult population and war veterans during 1972–2007. Sleep. (2013) 36:1041–50. 10.5665/sleep.2806 23814341 PMC3669062

[125] Nadorff MR Drapeau CW Pigeon WR. Psychiatric illness and sleep in older adults: comorbidity and ppportunities for intervention. Sleep Med Clin. (2018) 13:81–91. 10.1016/j.jsmc.2017.09.008 29412986

[126] Nadorff MR Nadorff DK Germain A. Nightmares: under-reported, undetected, and therefore untreated. J Clin Sleep Med. (2015) 11:747–50. 10.5664/jcsm.4850 25845898 PMC4481058

[127] Schredl M Seeking professional help for nightmares: a representative study. Eur J Psychiatry. (2013) 27:259–64. 10.4321/S0213-61632013000400004

[128] Thünker J Norpoth M Aspern M Kapanci T Pietrowsky R Nightmares: knowledge and attitudes in health care providers and nightmare sufferers. J Public Health Epidemiol. (2014) 6:223–8. 10.5897/JPHE2013.0565

[129] Pagel JF. Nightmares and disorders of dreaming. Am Fam Physician. (2000) 61:2037–42. 10779247

[130] Levin R Nielsen TA. Disturbed dreaming, posttraumatic stress disorder, and affect distress: a review and neurocognitive model. Psychol Bull. (2007) 133:482–528. 10.1037/0033-2909.133.3.482 17469988

[131] Muntean ML Trenkwalder C Walters AS Mollenhauer B Sixel-Doring F. REM sleep behavioral events and dreaming. J Clin Sleep Med. (2015) 11:537–41. 10.5664/jcsm.4698 25665694 PMC4410927

[132] Bhat S Chokroverty S Kabak B Yang QR Rosen D. Dream-enacting behavior in non-rapid eye movement sleep. Sleep Med. (2012) 13:445–6. 10.1016/j.sleep.2011.10.029 22341904

[133] Zhang Y Ren R Sanford LD Yang L Ni Y Zhou J. The effects of prazosin on sleep disturbances in post-traumatic stress disorder: a systematic review and meta-analysis. Sleep Med. (2020) 67:225–31. 10.1016/j.sleep.2019.06.010 31972510 PMC6986268

[134] Alfonsi V D'Atri A Scarpelli S Mangiaruga A De Gennaro L. Sleep talking: a viable access to mental processes during sleep. Sleep Med Rev. (2019) 44:12–22. 10.1016/j.smrv.2018.12.001 30594004

[135] Oudiette D Dealberto MJ Uguccioni G Golmard JL Merino-Andreu M Tafti M Dreaming without REM sleep. Conscious Cogn. (2012) 21:1129–40. 10.1016/j.concog.2012.04.010 22647346

[136] Baldini T Loddo G Sessagesimi E Mignani F Cirignotta F Mondini S. Clinical features and pathophysiology of disorders of arousal in adults: a window into the sleeping brain. Front Neurol. (2019) 10:526. 10.3389/fneur.2019.00526 31164861 PMC6534078

[137] Uguccioni G Golmard JL de Fontreaux AN Leu-Semenescu S Brion A Arnulf I. Fight or flight? Dream content during sleepwalking/sleep terrors vs. rapid eye movement sleep behavior disorder. Sleep Med. (2013) 14:391–8. 10.1016/j.sleep.2013.01.014 23601752

[138] Antrobus J. REM and NREM sleep reports: comparison of word frequencies by cognitive classes. Psychophysiology. (1983) 20:562–8. 10.1111/j.1469-8986.1983.tb03015.x 6635096

[139] Foulkes D. Nonrapid eye movement mentation. Exp Neurol. (1967) 19(Suppl. 4):28–38. 10.1016/0014-4886(67)90154-9 6079780

[140] Zimmerman WB. Sleep mentation and auditory awakening thresholds. Psychophysiology. (1970) 6:540–9. 10.1111/j.1469-8986.1970.tb02243.x 4320882

[141] Solms M Dreaming and REM sleep are controlled by different brain mechanisms. Behav Brain Sci. (2000) 23:843–50; discussion 904–1121. 10.1017/S0140525X00003988 11515144

[142] Hobson JA Pace-Schott EF Stickgold R. Dreaming and the brain: toward a cognitive neuroscience of conscious states. Behav Brain Sci. (2000) 23:793–842; discussion 904–1121. 10.1017/S0140525X00003976 11515143

[143] LaBerge S Lucid dreaming: psychophysiological studies of consciousness during REM sleep. In: Bootzin RR Kihlstrom JF Schacter DL editors. Sleep and Cognition. Washington, DC: American Psychological Association (1990). p. 109–26.

[144] Dresler M Wehrle R Spoormaker VI Koch SP Holsboer F Steiger A. Neural correlates of dream lucidity obtained from contrasting lucid versus non-lucid REM sleep: a combined EEG/fMRI case study. Sleep. (2012) 35:1017–20. 10.5665/sleep.1974 22754049 PMC3369221

[145] Dresler M Eibl L Fischer CF Wehrle R Spoormaker VI Steiger A. Volitional components of consciousness vary across wakefulness, dreaming and lucid dreaming. Front Psychol. (2014) 4:987. 10.3389/fpsyg.2013.00987 24427149 PMC3877766

[146] Baird B Mota-Rolim SA Dresler M. The cognitive neuroscience of lucid dreaming. Neurosci Biobehav Rev. (2019) 100:305–23. 10.1016/j.neubiorev.2019.03.008 30880167 PMC6451677

[147] la Berge SP Nagel LE Dement WC Zarcone VP. Lucid dreaming verified by volitional communication during REM sleep. Percept Motor Skills. (1981) 52:727–32. 10.2466/pms.1981.52.3.727 24171230

[148] Sagnier S Coulon P Chaufton C Poli M Debruxelles S Renou P. Lucid dreams, an atypical sleep disturbance in anterior and mediodorsal thalamic strokes. Rev Neurol (Paris). (2015) 171:768–72. 10.1016/j.neurol.2015.08.005 26494569

[149] Lacaux C Izabelle C Santantonio G De Villele L Frain J Lubart T. Increased creative thinking in narcolepsy. Brain. (2019) 142:1988–99. 10.1093/brain/awz137 31143939

[150] Stumbrys T Erlacher D Schadlich M Schredl M. Induction of lucid dreams: a systematic review of evidence. Conscious Cogn. (2012) 21:1456–75. 10.1016/j.concog.2012.07.003 22841958

[151] Roehrs T Roth T. Sleep, sleepiness, sleep disorders and alcohol use and abuse. Sleep Med Rev. (2001) 5:287–97. 10.1053/smrv.2001.0162 12530993

[152] Schierenbeck T Riemann D Berger M Hornyak M. Effect of illicit recreational drugs upon sleep: cocaine, ecstasy and marijuana. Sleep Med Rev. (2008) 12:381–9. 10.1016/j.smrv.2007.12.004 18313952

[153] Vogel GW. A review of REM sleep deprivation. Arch Gen Psychiatry. (1975) 32:749–61. 10.1001/archpsyc.1975.01760240077006 165795

[154] Muzio JN Roffwarg HP Kaufman E. Alterations in the nocturnal sleep cycle resulting from LSD. Electroencephalogr Clin Neurophysiol. (1966) 21:313–24. 10.1016/0013-4694(66)90037-X 4162426

[155] Mota-Rolim SA Targino ZH Souza BC Blanco W Araujo JF Ribeiro S. Dream characteristics in a Brazilian sample: an online survey focusing on lucid dreaming. Front Hum Neurosci. (2013) 7:836. 10.3389/fnhum.2013.00836 24368900 PMC3857923

[156] Saunders DT Roe CA Smith G Clegg H. Lucid dreaming incidence: a quality effects meta-analysis of 50 years of research. Conscious Cogn. (2016) 43:197–215. 10.1016/j.concog.2016.06.002 27337287

[157] Snyder TJ Gackenbach J Individual differences associated with lucid dreaming. In: LaBerge S Gackenbach J editors. Conscious Mind, Sleeping Brain. New York, NY: Springer (1988). p. 221–59. 10.1007/978-1-4757-0423-5_10

[158] LaBerge S Levitan L Dement W Lucid dreaming: physiological correlates of consciousness during REM sleep. J Mind Behav. (1986) 7:251–8.

[159] Dane JR A Comparison of Waking Instructions and Posthypnotic Suggestion for Lucid Dream Induction. Atlanta: Georgia State University (1984).

[160] LaBerge SP Lucid Dreaming: An Exploratory Study of Consciousness During Sleep. Stanford: ProQuest Information & Learning (1980).

[161] la Berge SP Lucid dreaming as a learnable skill: a case study. Percept Motor Skills. (1980) 51:1039–42. 10.2466/pms.1980.51.3f.1039

[162] Siegel JM Tomaszewski KS Nienhuis R. Behavioral states in the chronic medullary and midpontine cat. Electroencephalogr Clin Neurophysiol. (1986) 63:274–88. 10.1016/0013-4694(86)90095-7 2419085 PMC9045735

[163] Villablanca JR de Andrés I Olmstead CE. Sleep-waking states develop independently in the isolated forebrain and brain stem following early postnatal midbrain transection in cats. Neuroscience. (2001) 106:717–31. 10.1016/S0306-4522(01)00329-3 11682158

